# Human norovirus GII.4 Hong Kong variant shares common ancestry with GII.4 Osaka and emerged in Thailand in 2016

**DOI:** 10.1371/journal.pone.0256572

**Published:** 2021-08-23

**Authors:** Watchaporn Chuchaona, Jira Chansaenroj, Jiratchaya Puenpa, Sarawut Khongwichit, Sumeth Korkong, Sompong Vongpunsawad, Yong Poovorawan

**Affiliations:** Center of Excellence in Clinical Virology, Faculty of Medicine, Chulalongkorn University, Bangkok, Thailand; University of Illinois at Chicago College of Medicine, UNITED STATES

## Abstract

Human norovirus is a leading cause of non-bacterial acute gastroenteritis, which affects all age groups and are found globally. Infections are highly contagious and often occur as outbreaks. Periodic emergence of new strains are not uncommon and novel variants are named after the place of first reported nucleotide sequence. Here, we identified human norovirus GII.4 Hong Kong variant in stool samples from Thai patients presented with acute gastroenteritis. Comparison of amino acid residues deduced from the viral nucleotide sequence with those of historical and contemporary norovirus GII.4 strains revealed notable differences, which mapped to the defined antigenic sites of the viral major capsid protein. Time-scaled phylogenetic analysis suggests that GII.4 Hong Kong shared common ancestry with GII.4 Osaka first reported in 2007, and more importantly, did not evolve from the now-prevalent GII.4 Sydney lineage. As circulation of norovirus minor variants can lead to eventual widespread transmission in susceptible population, this study underscores the potential emergence of the GII.4 Hong Kong variant, which warrants vigilant molecular epidemiological surveillance.

## Introduction

Human noroviruses are the most common cause of epidemic and sporadic acute gastroenteritis in all age groups [1]. Most outbreaks occur in closed community settings including schools, childcare facilities, restaurants, and hospitals [2]. There are as many as ten norovirus genogroups (GI to GX) and 48 genotypes [3], of which GII.4 genotype is most often detected in viral gastroenteritis patients. Reinfection throughout one’s lifetime is possible due to the emergence of new variants resulting from frequent viral mutation and genome recombination near the RNA-dependent RNA polymerase (RdRp) and the major capsid protein (VP1) genes [4]. Thus, the evolving genomic sequences in norovirus can potentially result in the viral escape from pre-existing immunity, and newly emergent variants may lead to increased incidence of norovirus infections worldwide [5].

VP1 is the major component of the capsid and the primary determinant of the viral structure [6, 7]. It is comprised of the shell (S) and the protruding (P) domains. The P domain interacts with the histo-blood group antigens (attachment molecules on the surface of the host cell) and is subjected to the host neutralizing antibody recognition [8–10]. Changes on the variable antigenic sites primarily on the surface of the P domain give rise to the emergence of new norovirus variant strains.

Over 70% of all globally reported circulating GII.4 norovirus strains over the past two decades have included at least 11 variant strains, of which six have caused pandemics (US 95–96, Farmington Hills 2002, Hunter 2004, Den Haag 2006b, New Orleans 2009, and Sydney 2012 [3, 11]. A study has shown that such variants are presumed to circulate at low levels long before they emerge as pandemic strains to cause widespread outbreaks [12]. One such variant recently under surveillance, GII.4 Hong Kong, was first identified among hospitalized patients in The Philippines beginning in 2017 and subsequently detected elsewhere in Asia and in Europe [13]. This prompted us to re-evaluate several unusual GII.4 strains circulating in Bangkok within the past five years. Here, we report the identification and characterization of two strains of GII.4 Hong Kong variant, which first emerged in 2016.

## Materials and methods

### VP1 gene amplification and genotyping

Archived complementary DNA samples from our previous enteric virus studies in Thailand [14, 15] were subjected to PCR to amplify partial RdRp and VP1 region with using previously described conditions [16]. The complete VP1 genes were amplified using norovirus-specific primers in S1 Table [17]. In this study, we nucleotide sequenced two strains of GII.4 Hong Kong variant (B2717 and B2793) from 2016 and 32 strains of GII.4 Sydney from 2017 to 2019. PCR products were resolved by agarose gel electrophoresis followed by gel-extraction purification (GeneAll Biotechnology, Seoul, Korea). After Sanger sequencing, nucleotide sequences were aligned using ClustalW, assembled using BioEdit version 7.2.0 [18], and subjected to an online norovirus genotyping tool (http://www.rivm.nl/mpf/norovirus/typingtool) [19]. Sequences were deposited in the GenBank database under the accession numbers MW521097-MW521130.

### Phylogenetic and Bayesian evolutionary analysis

B2717 and B2793 were compared to different norovirus GII.4 variants whose nucleotide sequences were available in the GenBank database. Phylogenetic tree was constructed using the maximum-likelihood method with 1,000 bootstrap replicates implemented in MEGA7 software [20]. For the evolutionary analysis of the GII.4 complete VP1 gene, we constructed time-measured phylogenetic analysis by using the Bayesian Markov Chain Monte Carlo (MCMC) method implemented in Bayesian Evolutionary Analysis Sampling Trees (BEAST) version 2.4.3 [21]. Nucleotide variations within and between clusters were examined by applying the maximum likelihood based on the Tamura-Nei 93 (TN93) nucleotide substitution model. Bayesian coalescent skyline tree prior used in this study assumed that each parameter value occurs in the distribution proportionally to the possibility of it occurring in the natural population. The algorithm constructed a distribution of parameters estimated from the selected simulations. Dataset was estimated as a tree prior with a chain length of 300 million and the Relaxed Clock Log-Normal model that allowed evolutionary rates to vary between clades. The calculated Effective Sample Size (ESS) values (greater than 200) was implemented in TRACER version 1.6 program (http://tree.bio.ed.ac.uk/software/tracer/). Analyzed plots were visualized with the 95% highest posterior density (HPD) intervals.

### Nucleotide, protein, and structural analysis

Pairwise nucleotide sequence similarity analysis between B2717 and 14 reference GII.4 sequences available from the GenBank database were assessed by using SimPlot version 3.5.1 [22]. Similarity plot was generated with a window size and step size of 200 and 20, respectively. Kimura 2-parameter distance model was used. The ratio of transitions and transversions was determined empirically. Three-dimensional crystal structure of the dimeric P domain of norovirus GII.4 (TCH05, Protein Data Bank accession number 3SKB) [23] served as template for visualizing amino acid residue changes identified in this study. Residues were mapped onto the structure using PyMOL version 1.3.

## Results

**Fig 1:**
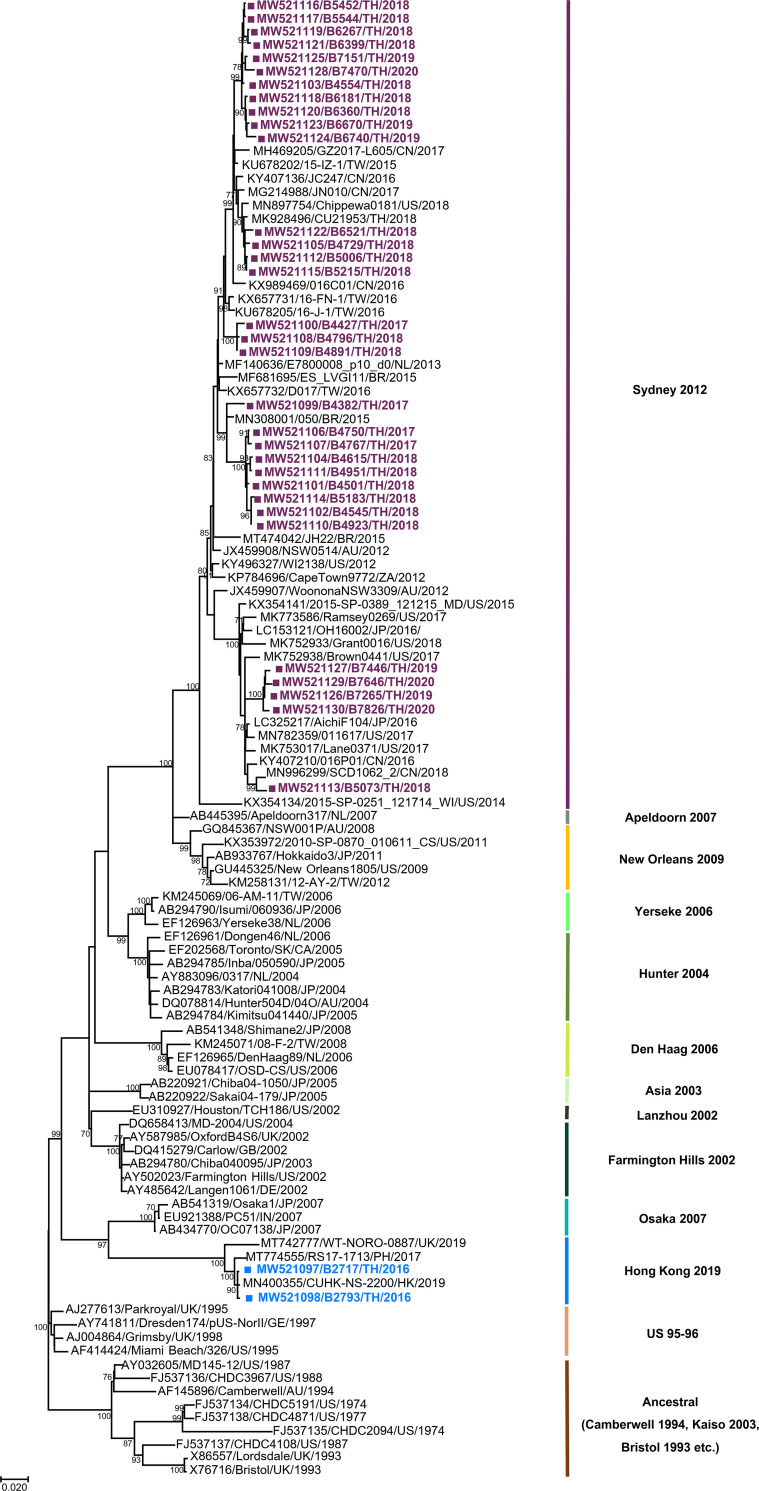
Phylogenetic tree of the complete VP1 sequences of the representative norovirus GII.4 variant (pandemic and non-pandemic) strains. Trees were generated using the maximum likelihood method from an alignment of the VP1 gene of 77 GII.4 reference (shown in black with accession number) and 34 strains identified in Thailand (denoted with squares). Scale bar represents nucleotide substitutions per site. Bootstrap values ≥70 are indicated at the nodes.

Due to the continual emergence of novel norovirus strains, which necessitated the recent re-classification of several norovirus genotypes [3], we initially re-examined the partial VP1 genes from several human norovirus GII.4 Sydney strains identified in Thailand from 2015–2017 [14]. Sequences were analyzed using the updated online norovirus genotyping tool, from which two Thai norovirus strains (designated B2717 and B2793) previously classified as GII.4 Sydney were reclassified as GII.4 Hong Kong variants. To confirm our findings, we amplified the complete VP1 gene from B2717 and B2793 and evaluated them together with 32 additional GII.4 Sydney strains identified since 2017 against the different GII.4 reference strains (Fig 1). As expected, both Thai norovirus GII.4 Hong Kong variants from 2016 grouped into the previously reported 2017–2019 GII.4 Hong Kong with nucleotide sequence identity of 96.4–99.8% and amino acid sequence identity of 97.2–99.8%. The lineage of GII.4 Hong Kong appears closest to that of GII.4 Osaka with ~90% nucleotide and ~92% amino acid sequence identity. In comparison, the nucleotide and amino acid sequence identity among the Thai and reference GII.4 Sydney strains ranged from 93.6–100% and 96.4–100%, respectively.

**Fig 2:**
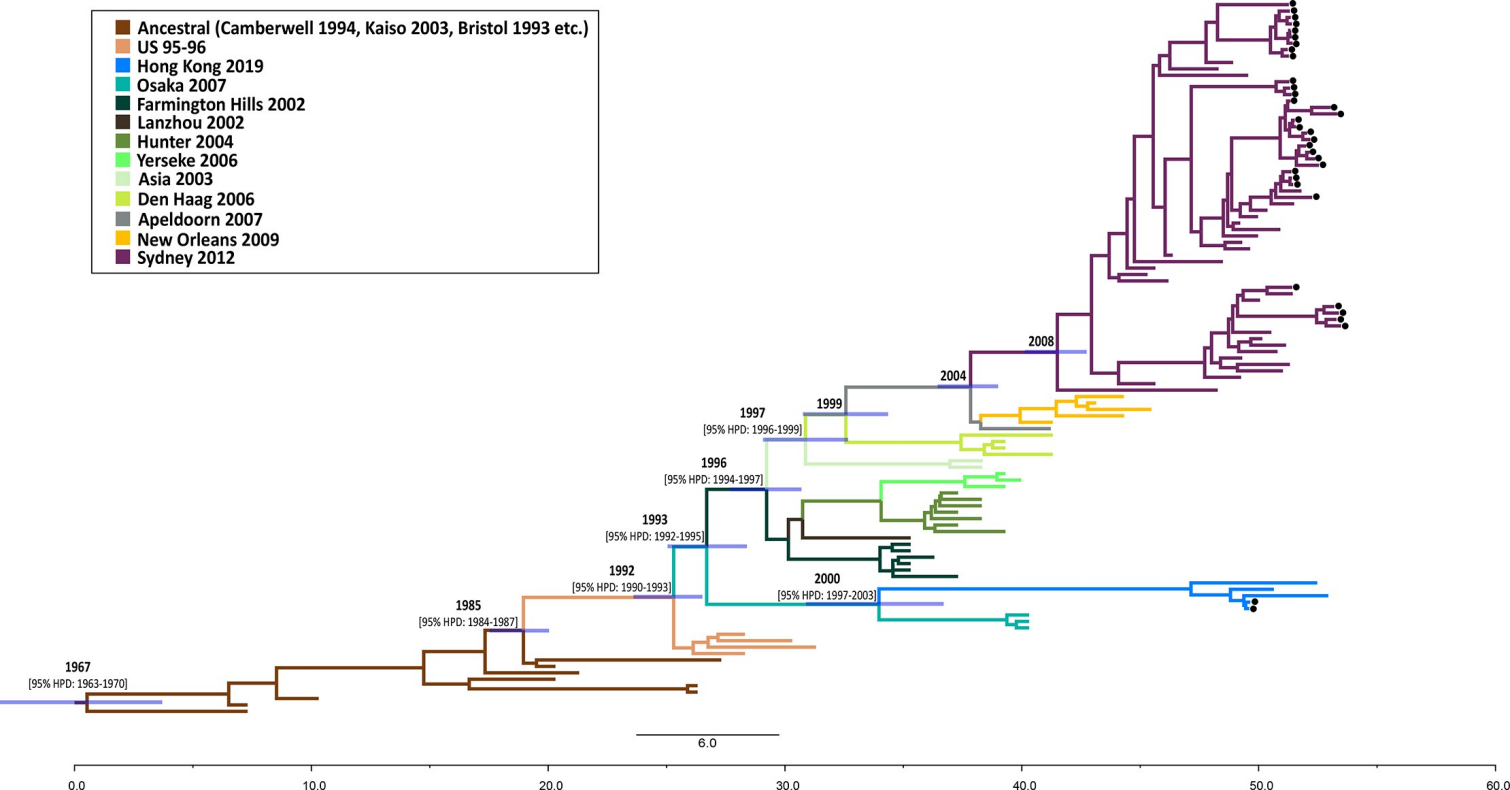
Bayesian time-scale phylogenetic analysis of norovirus GII.4 variants based on their complete VP1 sequences (n = 111). The 34 Thai GII.4 strains from this study including two Thai GII.4 Hong Kong (denote by dots) and the color-coded reference strains reported over the past 50 years are shown. The maximum clade credibility tree was constructed using the TreeAnnotator/FigTree. Bars at the branch nodes denote 95% highest posterior density intervals for the estimated year of divergence (bolded). Time scale is shown at the bottom of the tree (reference length represents 6 years).

To determine the evolutionary origin of the GII.4 Hong Kong variant, we next performed time-scaled phylogenetic tree using Bayesian Skyline Plot inference. The tree affirms that GII.4 Hong Kong was most closely related to GII.4 Osaka lineage, which first emerged in 2007 (Fig 2). Results further suggest that GII.4 Hong Kong diverged relatively early from a common ancestor, which gave rise to various GII.4 progenies, and did not evolve from the commonly prevalent GII.4 Sydney cluster of recent years. The overall evolutionary rate of the GII.4 capsid sequences was estimated to be 4.5×10^−3^ substitutions/site/year (95% HPD Intervals, 4.0×10^−3^–5.1×10^−3^).

**Fig 3:**
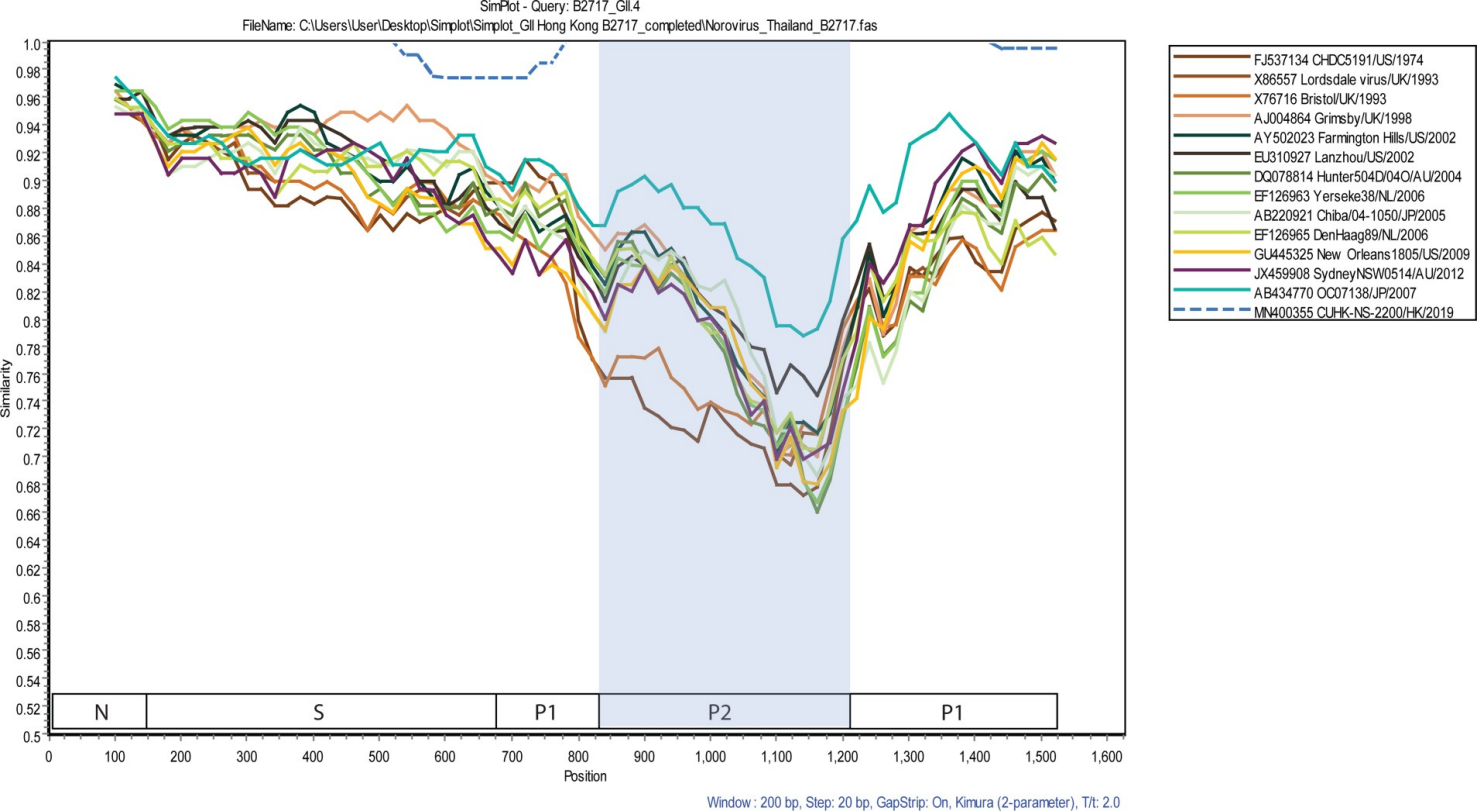
Pairwise nucleotide similarities between the complete VP1 sequence of the Thai GII.4 Hong Kong (B2717) and those of 14 other pandemic and non-pandemic GII.4 reference strains (shown in different colors). Analysis was performed using SimPlot program version 3.5.1 with B2717 as a query strain. N = amino-terminal, S = shell, and protruding domains P1 and P2.

SimPlot nucleotide pairwise analysis of the VP1 gene of B2717 showed near identity with the GII.4 Hong Kong (strain CUHK-NS-2200/HK/2019, accession number MN400355), but marked hypervariability with those of various GII.4 reference strains especially near the P2 region (S domain 85–96%, P1 domain 74–95%, P2 domain 66–91%) (Fig 3). Again, GII.4 Hong Kong appears most similar to GII.4 Osaka (strain Hu/OC07138/07/JP, accession number AB434770) even in the P2 domain (78–91% pairwise nucleotide identity) compared to other GII.4 lineages.

**Fig 4:**
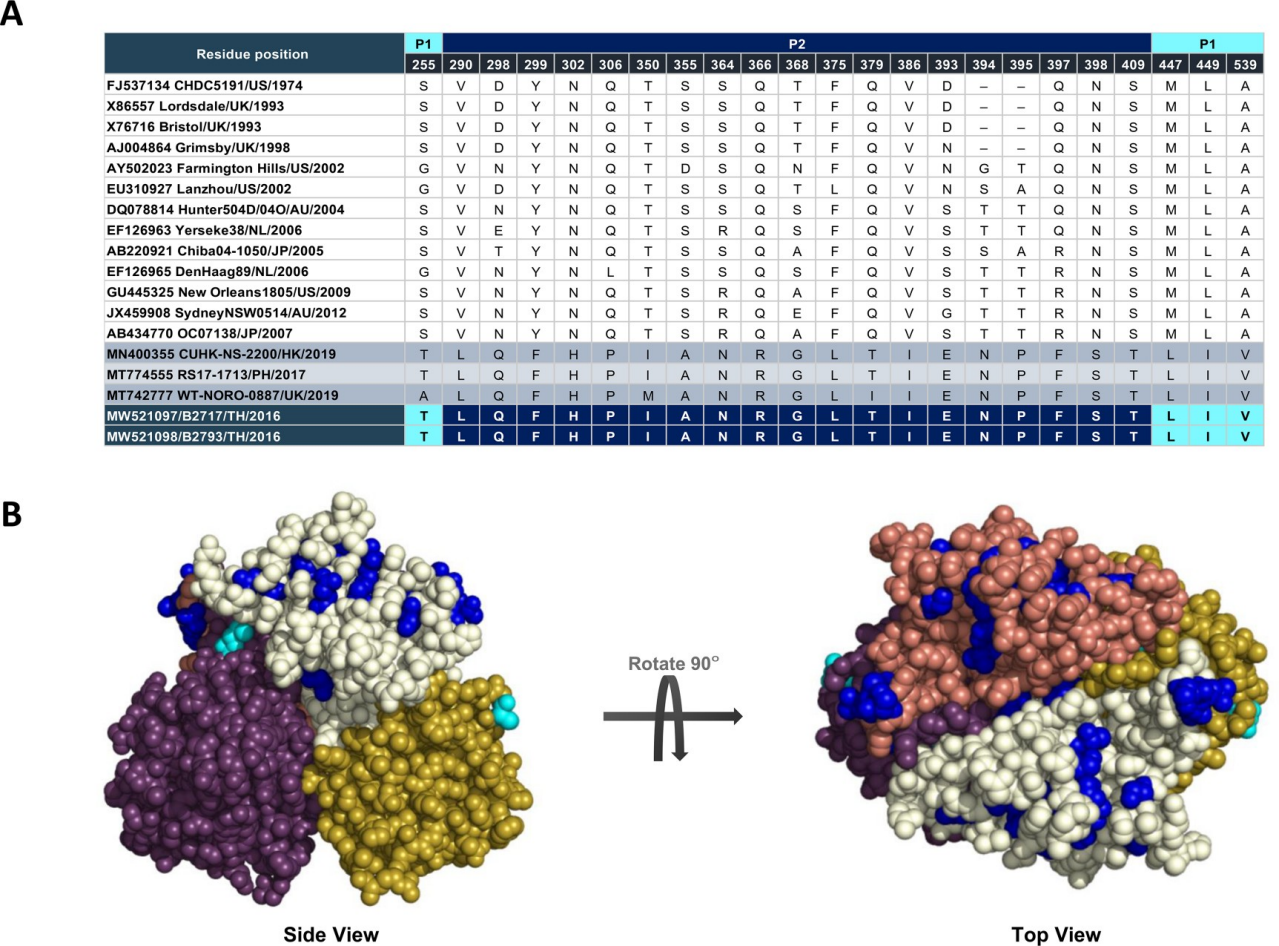
Amino acid residues on the major capsid protein VP1 of GII.4 Hong Kong. A) Amino acid differences in the P1 and P2 domains of the GII.4 Hong Kong compared with other pandemic and non-pandemic GII.4 VP1 sequences. The Thai GII.4 Hong Kong strains B2717 and B2793 are shown in the bottom two rows. B) Characteristic residues common to GII.4 Hong Kong were mapped onto the three-dimensional structure of the dimeric P domain (GII.4 Houston TCH05). Surface-exposed residue changes (light and dark blue) are shown on the P1 and P2 domain, respectively. Each P monomer is colored white/gold and pink/purple.

To identify prominent amino acid residues, which differentiate GII.4 Hong Kong from other recent GII.4 strains, we aligned VP1 protein sequence from different GII.4 lineages with the deduced amino acid residues from the VP1 gene sequences of B2717 and B2793 (Fig 4A). All GII.4 Hong Kong strains regardless of their origin appeared to share common residues at several positions. Most of the residues which were exclusive to the GII.4 Hong Kong strains were located on the P2 domain. They were 290L, 298Q, 299F, 302H, 306P, 355A, 364N, 366R, 368G, 375L, 386I, 393E, 394N, 395P, 397F, 398S and 409T (residue position and one-letter code). Distinctive residues in the P1 domain were 447L, 449I, and 539V. In the S domain, only 144V set GII.4 Hong Kong apart from the other GII.4 strains.

We next mapped these important residue differences onto the three-dimensional structure of an epidemic GII.4 (TCH05) dimeric P domain for which crystal structures have been solved (Fig 4B). Remarkably, all of the above residues unique to GII.4 Hong Kong mapped to the solvent-exposed surface on the P dimer. Furthermore, a significant number of residues mapped to known and novel antigenic epitopes (designated A through G) refined through a large-scale genomic study [24]. For example, 298Q and 368G were located on antigenic site A, while 375L appeared on antigenic site C. Four nearly contiguous residues mapped to antigenic site D (393E, 394N, 395P, and 397F). Finally, 355A and 364N affected the recently proposed antigenic site G. Taken together, the novelty of residues exclusive to GII.4 Hong Kong strains suggests potential changes in the antigenic characteristics that warrant further investigation.

## Discussion

The circulation of GII.4 Hong Kong in Thailand in 2016 was unexpected and occurred earlier than previously reported in the literature [13]. The fact that the VP1 sequences of the two Thai strains were nearly identical to those of other GII.4 Hong Kong strains analyzed points to this variant’s relatively inconspicuous circulation prior to 2017, possibly due to their low frequency of detection.

Although GII.4 Hong Kong possessed numerous exclusive mutations and has already caused infection in Asia and Europe, its potential to become pandemic like GII.4 Sydney is uncertain. Its shared ancestry with the non-pandemic GII.4 Osaka, however, does not necessarily preclude its virulence since the previous pandemic GII.4 New Orleans emerged from the same ancestral lineage with the non-pandemic GII.4 Apeldoorn. Moreover, unique residues on GII.4 Hong Kong affecting important antigenic sites, including the previously described residues at positions 352, 355, 368, and 378 [13], as well as additional residues described in this study, could enable possible evasion of pre-existing host immunity.

Interestingly, all GII.4 Hong Kong including the Thai strains possess RdRp gene from GII.P31, which was commonly found in combination with GII.4 Sydney in Thai patients infected with norovirus in 2017–2018 [15]. The standard method of partial RdRp sequence amplification performed as part of a minimal norovirus genotyping allowed us to deduce residues 348 to 510 of RdRp in the B2717 and B2793. Alignment of the amino acid sequences in this region of GII.P31 from this study, the prototypic strain (GenBank accession number AB434770), GII.4 Sydney (JX459908), and GII.4 Osaka (AB541319) revealed one unique and noteworthy difference in each variant. GII.P31 in Hong Kong, Sydney, and Osaka possessed P399I, S427F, and A360G, respectively. These changes are located in the palm and thumb subdomains of the norovirus RdRp, but none mapped to any RdRp motifs responsible for interaction with viral RNA template and RNA synthesis [25]. There is an increased awareness and recognition that novel norovirus variants emerged not only from changes in the VP1 capsid protein, but also the functional gain afforded by the new genotype of RdRp protein through recombination at the ORF1/ORF2 junction, or mutations in the RdRp, or both [26, 27]. Attention in determining important nucleotide regions of RdRp may be needed as part of a standard norovirus characterization in addition to the entire VP1 gene sequence in order to better determine changes in the RdRp, which may influence nucleotide incorporation speed, processivity, fidelity, and ultimately viral transmissibility.

With the increased public health measures brought on as a result of the recent global coronavirus pandemic, such as school closures, social distancing, and awareness in hygiene practice of frequent handwashing, a number of countries have reported decreases in the number of seasonal viral respiratory infections such as influenza [28, 29]. Even a decline in the incidence of norovirus outbreaks in several U.S. states has been associated with the implementation of coronavirus public health measures [30]. Therefore, modified human behaviors (such as better hygiene awareness, avoidance of crowded conditions, and other disease avoidance lifestyle changes) may potentially delay or reduce the magnitude of the next global pandemic norovirus outbreaks.

Surveillance and awareness of emergent human noroviruses, especially those belonging to the predominant GII.4 lineage, will require vigilance in molecular epidemiology and collaborative public health efforts. The global norovirus surveillance network for pediatric gastroenteritis NoroSurv (https://www.norosurv.org) led by the U.S. Centers for Disease Control and Prevention is one such example [31]. The collective norovirus sequence data could therefore potentially help monitor geographical trends in norovirus emergence and spread, as well as serve as a gold mine towards the development of the best vaccine candidates most likely to protect against circulating global norovirus strains.

## Conclusions

Similar to other RNA viruses, human norovirus variants periodically emerged to cause outbreaks worldwide. Here, we report the detection and characterization of the norovirus GII.4 Hong Kong variant, which first emerged in Thailand in 2016 and earlier than previously thought. The retrospective analysis of viral sequence data can reveal unexpected findings and help contribute to a more accurate timeline of the temporal and geographical emergence of virus variants.

## Supporting information

S1 Table Primers used to amplify the complete VP1 gene of GII.4 norovirus in this study. (DOCX)

## References

[1] Ahmed SM, Hall AJ, Robinson AE, Verhoef L, Premkumar P, Parashar UD, et al. Global prevalence of norovirus in cases of gastroenteritis: a systematic review and meta-analysis. Lancet Infect Dis. 2014; 14(8): 725–30. doi: 10.1016/S1473-3099(14)70767-4 24981041 PMC8006533

[2] Hall AJ, Wikswo ME, Manikonda K, Roberts VA, Yoder JS, Gould LH. Acute gastroenteritis surveillance through the National Outbreak Reporting System, United States. Emerg Infect Dis. 2013; 19(8): 1305–9. doi: 10.3201/eid1908.130482 23876187 PMC3739540

[3] Chhabra P, de Graaf M, Parra GI, Chan MC, Green K, Martella V, et al. Updated classification of norovirus genogroups and genotypes. J Gen Virol. 2019; 100(10): 1393–406. doi: 10.1099/jgv.0.001318 31483239 PMC7011714

[4] Bull RA, White PA. Mechanisms of GII.4 norovirus evolution. Trends Microbiol. 2011; 19(5): 233–40. doi: 10.1016/j.tim.2011.01.002 21310617

[5] Siebenga JJ, Vennema H, Zheng DP, Vinjé J, Lee BE, Pang XL, et al. Norovirus illness is a global problem: emergence and spread of norovirus GII.4 variants, 2001–2007. J Infect Dis. 2009; 200(5): 802–12. doi: 10.1086/605127 19627248

[6] Prasad BV, Hardy ME, Dokland T, Bella J, Rossmann MG, Estes MK. X-ray crystallographic structure of the Norwalk virus capsid. Science. 1999; 286(5438): 287–90. doi: 10.1126/science.286.5438.287 10514371

[7] Jung J, Grant T, Thomas DR, Diehnelt CW, Grigorieff N, Joshua-Tor L. High-resolution cryo-EM structures of outbreak strain human norovirus shells reveal size variations. Proc Natl Acad Sci U S A. 2019; 116(26): 12828–32. doi: 10.1073/pnas.1903562116 31182604 PMC6601263

[8] Choi JM, Hutson AM, Estes MK, Prasad BV. Atomic resolution structural characterization of recognition of histo-blood group antigens by Norwalk virus. Proc Natl Acad Sci U S A. 2008; 105(27): 9175–80. doi: 10.1073/pnas.0803275105 18599458 PMC2453692

[9] Koromyslova AD, Morozov VA, Hefele L, Hansman GS. Human norovirus neutralized by a monoclonal antibody targeting the histo-blood group antigen pocket. J Virol. 2019; 93(5): e02174–18. doi: 10.1128/JVI.02174-18 30541855 PMC6384083

[10] Ford-Siltz LA, Wales S, Tohma K, Gao Y, Parra GI. Genotype-specific neutralization of norovirus is mediated by antibodies against the protruding domain of the major capsid protein. J Infect Dis. 2020: jiaa116. doi: 10.1093/infdis/jiaa116 32179892

[11] Hoa Tran TN, Trainor E, Nakagomi T, Cunliffe NA, Nakagomi O. Molecular epidemiology of noroviruses associated with acute sporadic gastroenteritis in children: global distribution of genogroups, genotypes and GII.4 variants. J Clin Virol. 2013; 56(3): 185–93. doi: 10.1016/j.jcv.2012.11.011 23218993

[12] Ruis C, Lindesmith LC, Mallory ML, Brewer-Jensen PD, Bryant JM, Costantini V, et al. Preadaptation of pandemic GII.4 noroviruses in unsampled virus reservoirs years before emergence. Virus Evol. 2020; 6(2): veaa067. doi: 10.1093/ve/veaa067 33381305 PMC7751145

[13] Chan MC, Roy S, Bonifacio J, Zhang LY, Chhabra P, Chan JCM, et al. Detection of Norovirus Variant GII.4 Hong Kong in Asia and Europe, 2017–2019. Emerg Infect Dis. 2021; 27(1): 289–93. doi: 10.3201/eid2701.203351 33350912 PMC7774557

[14] Thanusuwannasak T, Puenpa J, Chuchaona W, Vongpunsawad S, Poovorawan Y. Emergence of multiple norovirus strains in Thailand, 2015–2017. Infect Genet Evol. 2018; 61: 108–12. doi: 10.1016/j.meegid.2018.03.021 29597056

[15] Chuchaona W, Chansaenroj J, Wanlapakorn N, Vongpunsawad S, Poovorawan Y. Recombinant GII.Pe-GII.4 Norovirus, Thailand, 2017–2018. Emerg Infect Dis. 2019; 25(8): 1612–4. doi: 10.3201/eid2508.190365 31310212 PMC6649319

[16] Phumpholsup T, Chieochansin T, Vongpunsawad S, Vuthitanachot V, Payungporn S, Poovorawan Y. Human norovirus genogroup II recombinants in Thailand, 2009–2014. Arch Virol. 2015; 160(10): 2603–9. doi: 10.1007/s00705-015-2545-5 26215446

[17] Debbink K, Costantini V, Swanstrom J, Agnihothram S, Vinjé J, Baric R, et al. Human norovirus detection and production, quantification, and storage of virus-like particles. Curr Protoc Microbiol. 2013; 31: 15K.1.1–45. doi: 10.1002/9780471729259.mc15k01s31 24510290 PMC3920292

[18] Hall TA. BioEdit: A user-friendly biological sequence alignment editor and analysis program for Windows 95/98/NT. Nucl Acids Symp Ser. 1999; 41: 95–8.

[19] Kroneman A, Vennema H, Deforche K, v d Avoort H, Peñaranda S, Oberste MS, et al. An automated genotyping tool for enteroviruses and noroviruses. J Clin Virol. 2011; 51(2): 121–5. doi: 10.1016/j.jcv.2011.03.006 21514213

[20] Kumar S, Stecher G, Tamura K. MEGA7: Molecular Evolutionary Genetics Analysis Version 7.0 for Bigger Datasets. Mol Biol Evol. 2016; 33(7): 1870–4. doi: 10.1093/molbev/msw054 27004904 PMC8210823

[21] Bouckaert R, Heled J, Kühnert D, Vaughan T, Wu CH, Xie D, et al. BEAST 2: a software platform for Bayesian evolutionary analysis. PLoS Comput Biol. 2014; 10(4): e1003537. doi: 10.1371/journal.pcbi.1003537 24722319 PMC3985171

[22] Lole KS, Bollinger RC, Paranjape RS, Gadkari D, Kulkarni SS, Novak NG, et al. Full-length human immunodeficiency virus type 1 genomes from subtype C-infected seroconverters in India, with evidence of intersubtype recombination. J Virol. 1999; 73(1): 152–60. doi: 10.1128/JVI.73.1.152-160.1999 9847317 PMC103818

[23] Shanker S, Choi JM, Sankaran B, Atmar RL, Estes MK, Prasad BV. Structural analysis of histo-blood group antigen binding specificity in a norovirus GII.4 epidemic variant: implications for epochal evolution. J Virol. 2011; 85(17): 8635–45. doi: 10.1128/JVI.00848-11 21715503 PMC3165782

[24] Tohma K, Lepore CJ, Gao Y, Ford-Siltz LA, Parra GI. Population Genomics of GII.4 Noroviruses Reveal Complex Diversification and New Antigenic Sites Involved in the Emergence of Pandemic Strains. mBio. 2019; 10(5): e02202–19. doi: 10.1128/mBio.02202-19 31551337 PMC6759766

[25] Deval J, Jin Z, Chuang YC, Kao CC. Structure(s), function(s), and inhibition of the RNA-dependent RNA polymerase of noroviruses. Virus Res. 2017; 234: 21–33. doi: 10.1016/j.virusres.2016.12.018 28041960 PMC7114559

[26] Parra GI. Emergence of norovirus strains: A tale of two genes. Virus Evol. 2019; 5(2): vez048. doi: 10.1093/ve/vez048 32161666 PMC6875644

[27] Ruis C, Roy S, Brown JR, Allen DJ, Goldstein RA, Breuer J. The emerging GII.P16-GII.4 Sydney 2012 norovirus lineage is circulating worldwide, arose by late-2014 and contains polymerase changes that may increase virus transmission. PLoS One. 2017; 12(6): e0179572. doi: 10.1371/journal.pone.0179572 28662035 PMC5491022

[28] Soo RJJ, Chiew CJ, Ma S, Pung R, Lee V. Decreased Influenza Incidence under COVID-19 Control Measures, Singapore. Emerg Infect Dis. 2020; 26(8):1933–5. doi: 10.3201/eid2608.201229 32339092 PMC7392467

[29] Wong NS, Leung CC, Lee SS. Abrupt Subsidence of Seasonal Influenza after COVID-19 Outbreak, Hong Kong, China. Emerg Infect Dis. 2020; 26(11): 2753–5. doi: 10.3201/eid2611.200861 32852264 PMC7588551

[30] Kraay ANM, Han P, Kambhampati AK, Wikswo ME, Mirza SA, Lopman BA. Impact of nonpharmaceutical interventions for severe acute respiratory syndrome coronavirus 2 on norovirus outbreaks: An analysis of outbreaks reported by 9 US states. J Infect Dis. 2021; 224(1): 9–13. doi: 10.1093/infdis/jiab093 33606027 PMC7928764

[31] Cannon JL, Bonifacio J, Bucardo F, Buesa J, Bruggink L, Chan MC, et al. Global Trends in Norovirus Genotype Distribution among Children with Acute Gastroenteritis. Emerg Infect Dis. 2021; 27(5): 1438–45. doi: 10.3201/eid2705.204756 33900173 PMC8084493

