# Supplementary material for: Human norovirus GII.4 Hong Kong variant shares common ancestry with GII.4 Osaka and emerged in Thailand in 2016
Source: PLoS One. 2021 Aug 23;16(8):e0256572. doi: 10.1371/journal.pone.0256572 (PMC8382166; doi:10.1371/journal.pone.0256572)
Supplement: S1 Table — (DOCX) [file pone.0256572.s001.docx]

**S1 Table**. Primers used to amplify the complete VP1 gene of GII.4 norovirus in this study.

| **Primer** | **Sense** | **Sequence (5’-3’)** | **Position** |
| --- | --- | --- | --- |
| HNoV_F_RdRp_5021-5043 | F | ATG TTC AGA TGG ATG AGR TTC TC | 5012-5034 |
| GII4VP1_R1cu | R | GGT TTR GTT CTT GAY TCA ACT GT | 5739-5761 |
| GII4VP1_F1cu | F | TAG CAA TGY TGT AYA CAC CAC T | 5620-5641 |
| GII4VP1_R2cu | R | TCC AAR TYC ATG TTG GGR TAC C | 6412-6433 |
| GII4VP1_F3cu | F | AYG AAC CCC AAC ART GGG TGC T | 6277-6298 |
| SBV2_180R | R | GCT TGG ARC ATC TCY TTR TCA TG | 6881-6903 |
| PanGIIR* | R | GTC CAG GAG TCC AAA A | 7434-7449 |

F=forward, R=reverse.

Nucleotide position numbering based on Hu/Houston/TCH186/2002/US (accession number EU310927).

* Debbink K, Costantini V, Swanstrom J, Agnihothram S, Vinjé J, Baric R, et al. Human norovirus detection and production, quantification, and storage of virus-like particles. Curr Protoc Microbiol. 2013; 31: 15K.1.1-45. https://doi.org/10.1002/9780471729259 .mc15k01s31 PMID: 24510290 (From Reference 17)
